# Optimizing Test and Treat in Malawi: health care worker perspectives on barriers and facilitators to ART initiation among HIV-infected clients who feel healthy

**DOI:** 10.1080/16549716.2020.1728830

**Published:** 2020-02-26

**Authors:** Kathryn Dovel, Khumbo Phiri, Misheck Mphande, Deborah Mindry, Esnart Sanudi, Mcdaphton Bellos, Risa M. Hoffman

**Affiliations:** a Division of Infectious Diseases, David Geffen School of Medicine, University of California Los Angeles (UCLA), Los Angeles, CA, USA; b Partners in Hope Medical Centre, EQUIP Innovations for Health, Lilongwe, Malawi; c UC Global Health Institute, Center for Women’s Health Gender and Empowerment, Los Angeles, CA, USA

**Keywords:** Universal treatment, Test and Treat, early ART initiation, sub-Saharan Africa, healthy populations, barriers to care

## Abstract

**Background**: Test and Treat has been widely adopted throughout sub-Saharan Africa, whereby all HIV-positive individuals initiate antiretroviral therapy (ART) immediately upon diagnosis and continue for life. However, clients who feel healthy may delay ART initiation, despite being eligible under new treatment guidelines.

**Objective**: We examined health care worker (HCW) perceptions and experiences on how feeling healthy positively or negatively influences treatment initiation among HIV-positive clients in Malawi.

**Methods**: We conducted 12 focus group discussions with 101 HCWs across six health facilities in Central Malawi. Data were analyzed through constant comparison methods using Atlas.ti7.5.

**Results**: Feeling healthy influences perceptions of ART initiation among HIV-positive clients. HCWs described that healthy clients feel that there are few tangible benefits to immediate ART initiation, but numerous risks. Fear of stigma and unwanted disclosure, disruption of daily activities, fear of side effects, and limited knowledge about the benefits of early initiation were perceived by HCWs to deter healthy clients from initiating ART.

**Conclusion**: Feeling healthy may exacerbate barriers to ART initiation. Strategies to reach healthy clients are needed, such as chronic care models, differentiated models of care that minimize disruptions to daily activities, and community sensitization on the benefits of early initiation.

## Background

Early initiation of antiretroviral therapy (ART) is critical in order to decrease morbidity and mortality among individuals living with HIV, and to prevent further HIV transmission [1,2]. With the rollout of new universal treatment policies (i.e., Test and Treat), all individuals living with HIV can now initiate ART immediately and continue for life, regardless of CD4 count or WHO clinical (parameters used to measure stages of chronic HIV infection) [3,4]. Test and Treat policies have been widely adopted throughout sub-Saharan Africa [5] and are anticipated to facilitate early ART initiation by streamlining access to HIV treatment [3].

However, access to treatment alone may not improve early ART initiation among asymptomatic individuals who feel healthy (hereafter referred to as ‘healthy clients’) [6]. Clients with higher CD4 counts (representing early stages of HIV) are less likely to initiate treatment than clients with lower CD4 counts [7–10]. In sub-Saharan Africa, previous changes in ART eligibility criteria did not increase early ART initiation rates, with the median CD4 count at time of initiation remaining stable for over a decade (2002–2013) [9].

Healthy individuals living with HIV may face additional barriers to ART initiation, limiting the impact of universal Test and Treat policies. Existing literature show that healthy clients may see little benefits to early ART initiation and instead, associate ART initiation with time and financial costs, as well as fear of unwanted disclosure and side effects [11–14]. However, only a handful of studies on ART barriers have been conducted after the implementation of universal Test and Treat [12,13]. Literature from Option B+, a policy that has provided universal treatment for pregnant/breastfeeding women since 2016, found unique barriers to ART initiation among healthy women, including concerns about partner support, feeling healthy, poor knowledge about early ART initiation, needing time to accept their new status, and fear of side effects [15–18]. Additional information is needed to understand how ART initiation barriers may be similar or different for healthy clients under new universal Test and Treat policies compared to barriers described under prior guidelines that used clinical staging to determine ART eligibility.

In this study, we utilized focus group discussions with health care workers (HCWs) to understand HCW perspectives on early ART initiation for healthy clients under the new Test and Treat policy in Malawi. We included multiple cadres of HCWs, including ART providers and support staff who conduct HIV testing, in-depth counseling, and community-based tracing activities for HIV-positive individuals who miss follow-up appointments. We draw from a practice-based evidence framework that highlights the importance of understanding the experiences and practices of HCWs in order to develop more effective health interventions [19]. Perspectives from HCWs are particularly important since they regularly interact with clients, and have the ability to compare and contrast barriers to ART initiation experienced by healthy versus ill clients.

## Methods

### Study setting

HIV prevalence among adults (15–49 years) in Malawi is approximately 9% [20]. In July 2016, Malawi implemented Test and Treat across all health facilities [21]. The study was completed between 16 August and 14 October 2016 at 6 ART clinics in central Malawi that represent a diverse range of health facilities (Table 1). Two sites offered same-day ART initiation and four offered ART initiation after completing group counseling and presenting with a guardian (i.e., a trusted family member or friend who is identified to provide support for the individual’s HIV treatment plan and/or pick-up ART prescriptions as needed).

**Table 1.** Facility characteristics

Facility	Facility Type	Facility Ownership	Location	District	ART cohort size
1	District Hospital	Government	Rural	Dowa	1174
2	Rural Hospital	Government	Peri – Urban	Dowa	1699
3	Health Centre	Government	Rural	Kasungu	658
4	Health Centre	Government	Rural	Kasungu	702
5	Mission Hospital	Private	Rural	Dowa	1549
6	Mission Hospital	Private	Rural	Lilongwe	1919

**Table 2.** Health care worker demographics

Description	n (%)
Health care worker position	
Provider	40 (40%)
Support staff	61 (60%)
Female	70 (70%)
Age	
18–34	53 (53%)
35–44	37 (37%)
>45	11 (11%)
# years working in HIV service delivery	
<1 year	3 (3%)
1–2 years	16 (16%)
>2 years	67 (67%)
Unknown	15 (15%)

### Data collection

We conducted 12 focus group discussions (FGDs) with HCWs who actively supported the delivery of HIV testing and treatment services at participating facilities (101 HCWs in total). FGDs were conducted separately with ART providers (defined as clinical staff who distributes ART; 40 providers in total) and support staff (defined as community health workers and HIV counselors; 61 support staff in total). FGDs were conducted in quiet, private locations at health facilities, and were led by two trained, local research assistants. The FGD guide included questions about barriers and facilitators to ART initiation under Test and Treat, perceived differences in barriers and facilitators for healthy versus ill clients, why healthy clients had (or not) different experiences with ART initiation as compared to ill clients. We define healthy clients as those who ‘feel healthy’ instead of the clinical definitions of health (asymptomatic or high CD4 count) because CD4 and WHO staging measures were removed from Malawi’s standard care with the rollout of Test and Treat [22].

FGDs were audio recorded in Chichewa, the local language, and lasted an average of 90 minutes. Audio recordings were transcribed verbatim and translated into English. Spot checks of FGD transcripts and translations were regularly conducted to ensure transcriptions were complete and translations were accurate.

### Analysis

Constant comparison methods were implemented using Atlas.ti7.5 [23]. KD, KP, and DM reviewed the transcripts and applied deductive and inductive codes. Codes were compared for consistency and differences were resolved. Deductive, apriori codes were developed from the existing literature. New themes and subthemes also emerged using a modified grounded theory approach [24]. Dominant themes mentioned across most FGDs were extracted and are presented using exact quotes to highlight each theme.

### Ethics

Ethical approval was attained by the Institutional Review Board at University of California Los Angeles and the National Health Sciences Review Committee in Malawi. Participants were not paid for participation but were given refreshments during the FGDs. Oral consent was provided. No identifiers were collected.

## Results

One hundred and one HCWs participated in a focus group discussion, including 40 ART providers and 61 support staff. Nearly all HCWs were under 45 years of age, had been working in HIV services for over 2 years, and 70% were female (Table 2).

HCWs described how, from their experiences, feeling healthy seemed to exacerbate known barriers to ART initiation. Providers reported six major barriers to ART initiation experienced by healthy clients: (1) fear of stigma; (2) fear of unwanted disclosure; (3) needing time to process and accept their new HIV status; (4) limited knowledge about the benefits of early ART initiation; (5) disruption of routine life; and (6) fear of side effects. Below we describe HCW reports about how feeling healthy interacts with each barrier.

### Fear of stigma

Health care workers described fear of community-based stigma as a dominant barrier to ART initiation for both healthy and ill clients. Stigma is primarily experienced through gossip within communities’, such as frequent discussions about individuals who were known or suspected HIV-positive.

The community discriminates [against] those [who are] HIV positive, and clients see this. So clients say, ‘Aah, if I start ART and these people find out [I am HIV-positive], I will experience exactly what they do to others [discrimination]. I should better not start ART.’ (Facility 4, Rural, Male, provider)

While fear of stigma is believed to be pervasive for all clients, healthy clients may lack the motivation needed to overcome this barrier. In contrast, HCWs described how the benefit of renewed health for ill clients may outweigh the cost of potential stigma that could be experienced when initiating ART.

It is hard for the person who is not sick [to initiate ART], while for the person who is sick it is not as hard because the person is already in pain and is seeking relief. So it is simple for the [sick] client to start ART. He has the mentality that his health will improve [after starting ART], unlike someone who is healthy who may think that it is not very important for him to start ART. (Facility 4 Rural, Male provider)

### Unwanted disclosure

Fear of unwanted disclosure due to accessing ART services is a dominant theme across all but one FGD. In Malawi, ART is often offered independently of other outpatient services, and only on particular days, making it clear to community members and other clients who are accessing ART. Being seen in an ART clinic results in immediate assumptions about one’s HIV status.

Because we only have one day per week when people can access ART, it is obvious that if one comes to the hospital on a Tuesday, it definitely means they are HIV-positive. The other issue is the benches [waiting spaces] here at the hospital. The HIV wing is known to everyone in the community so if one is seen sitting on any of the benches in the HIV wing, then people know for sure that this one has HIV. (Facility 4, Rural, Male provider)

A few support staff believe that clients also fear overt breaches in confidentiality by HCWs, although this was significantly less prominent.

Some of us do not know how to hold our tongue when we see a person getting on ART. We talk about it with others. So when the person defaults we ask him [why he defaulted] and he says that he had heard about his status from other people. (Facility 4, Rural, Male support staff)

HCWs believe that healthy clients are more concerned about unwanted disclosure than ill clients. For healthy clients, treatment initiation greatly increases risk of unwanted disclosure. This population does not have physical signs or symptoms that would otherwise raise suspicion of HIV. For them, attending ART appointments presents the greatest risk of unwanted disclosure.

People who look like they are healthy and still strong are afraid to disclose their new status. When they start ART, the community will know the place where they access treatment. When the community sees that they are at the facility frequently, the community will know that they now have HIV. So in order to hide their new status, clients would rather wait [to initiate]. Healthy clients choose to wait until they are sick. (Facility 5, Rural, Male support staff)

In contrast, ill clients may have less fear about being seen at the ART clinic because community members may already suspect a positive HIV status due to chronic poor health and multiple visits to the health facility prior to ART initiation.

I heard if someone has lost weight, people refer to him as ‘clothes hangers’. Some say people on ART work as phone credit, they always have to top up airtime to be able to use it. (Facility 3, Rural, Female supporting staff)

Most HCWs report that it is easier for ill clients to disclose their status because they often need assistance from friends and family members in order to regain health. It is not uncommon for sick adults to attend health facilities with a caregiver who helps with transport and navigation of health services. Caregivers are often involved in clients’ use of health services and home-based care. Disclosing one’s HIV status is considered necessary in order for caregivers to fully support clients’ recovery. In contrast, healthy clients can manage their own health care without support from others.

Sometimes the health worker will request that the sick patient disclose their status to their guardian since they will need special attention and special diet, but there is not a compelling reason for one who is healthy to disclose their status to anyone at all. (Facility 2, Rural, female provider)

A minority of HCWs acknowledge that early initiation could help healthy clients avoid unwanted disclosure by avoiding poor health in the future, although most HCWs believe clients often think about immediate repercussions of ART initiation, not future benefits.

Starting treatment while still healthy can also reduce discrimination because communities know that if the person looks like that [sick], it is HIV as HIV stories are everywhere in the villages. Everyone knows the sick person will be infected with HIV in one way or another. This [treatment] can reduce discrimination. (Facility 3, Urban, Male provider)

### Clients need time to accept HIV diagnosis

The majority of HCWs believe healthy clients need time to accept their HIV positive status before initiating ART. One provider reflects on the contrast between the time given for clients to accept one’s status in the pre- versus post-Test and Treat eras.

Previously when somebody was tested HIV-positive, he had to wait for CD4 count results which determined whether the person should start right way or wait until the CD4 is low. So that period gave room for the person to reflect on the results and make a decision to start ART. With Test and Treat, clients start ART while in the counselling process. The client is still in the process of healing and making a decision to start ART or not … (Facility 6, Rural, Male supporting staff)

### Disruption of routine life

Initiating ART requires regular interaction with the health system and altering daily routines. National guidelines require monthly ART appointments for the first 6-months of treatment. Health care workers perceive this disruption as an important barrier to care for healthy clients who would otherwise be able to continue life as usual.

The healthy person can`t afford to stand in a long queue for ART instead of working. So they stop taking ART altogether because they want to use the time they “waste” at the facility to do other things. (Facility 2, Rural, Female provider)

For healthy clients who are working or have large household responsibilities, continual clinic visits are especially disruptive without immediate benefits. In contrast, sick clients are understood to accept the inconvenience of clinic visits given the potential benefits of improved health and restoration of their routine life.

When someone is sick they have no option but to receive treatment. [They will do] whatever it is that will make them well, return to their normal health, and resume their duties. (Facility 2, Rural, Female provider)

### Fear of side effects

Health care workers report that side effects remain a major barrier to treatment, especially for healthy clients who may believe the risks of side effects outweigh the immediate benefits of ART initiation.

I think some people don’t start ART because they feel healthy and therefore they think that taking ART will bring some problems into their health. They are afraid of side effects. (Facility 4, Rural, Male support staff)

### Knowledge of ART benefits

Health care workers believe clients have limited knowledge about the benefits of early ART initiation.

Healthy clients think there is nothing that ART is going to do in their bodies since they are not ill. So they choose to start ART when they get ill and say ‘If I take ART then and get better, I will know that it’s because of ART”. (Facility 4, Rural, Male provider)

In addition, nearly all HCWs report that clients still perceive ART as treatment for illness, drawing on clients’ previous knowledge and experiences with old eligibility criteria that allowed initiation only for advanced clinical staging or a low CD4 cell count.

It is hard for a person to start ART fast before getting sick because back in the day people were not starting ART instantly so they still have that mentality. (Facility 4, Rural, Female support staff)

As a solution, HCWs suggest mass community sensitization campaigns in order to inform communities about the new Test and Treat policy and early initiation.

Providing information to the people before they come to the hospital is very crucial. This will ensure that people have knowledge of Test and Treat before testing for HIV and thus they will not have a difficulty initiating. (Facility 5, Rural, Female support staff)

In the absence of community sensitization, some providers suggest promoting sensitization through facility-based health talks. Large groups could be targeted, such as patients in general outpatient departments or adults attending clinics for children under five years of age.

In addition, we can also help spread this information by sharing it with our patients even in the outpatient department. When they come for assistance we should just take our time to explain one or two things about the Test and Treat program. Thus, slowly but surely the information will stick in their minds and also spread to communities. (Facility 1, Rural, Female provider)

## Discussion

This study contributes to the growing body of literature on barriers to early ART initiation. This is one of the first to examine HCW perceptions of early ART initiation under the new universal Test and Treat policy, and highlights the role of health on decisions about ART initiation. We find that barriers to ART initiation remain despite streamlined access to care under universal Test and Treat. Our findings support existing literature that clients who feel ill have clear and immediate benefits from treatment initiation, namely improving their poor health and allowing them to return to a sense of normalcy [25–29]. For healthy clients, however, the benefits are not as clear, and may not be strong enough to overcome the perceived risks associated with treatment initiation while healthy [14,30–33]. HCWs perceive that feeling healthy changes the meaning of ART initiation for clients, increasing risk of unintended consequences, especially unwanted disclosure among community and family members. A novel, important finding from this study is that feeling healthy is understood to interact with known barriers to care widely described under older treatment guidelines, exacerbating challenges to ART initiation [8,32,34–37]. Notably, there was little variation between ART providers and support staff – both cadres believe good health compounded the barriers to ART initiation that have been described under prior guidelines which used clinical staging to define treatment eligibility.

Fear of disclosure was the most commonly-cited barrier to initiation. The provider narrative suggests that fear of stigma leads healthy clients to avoid disclosure at all cost. Unwanted disclosure is frequent in sub-Saharan Africa where local communities use multiple strategies to make ‘educated guesses’ about individuals’ HIV status [26,38,39]. For healthy clients, attending HIV services is one of the primary ways through which their status may be disclosed since they do not exhibit physical symptoms that raise community suspicion [34]. Our findings support the critical importance of private HIV services [30,32]. Early ART initiation may be more acceptable if private, confidential HIV services are available [33].

Simple solutions to improve privacy and decrease fear of unwanted disclosure include private waiting spaces for HIV services and allowing clients to initiate ART at facilities outside their local community. More complex solutions include complete integration of HIV services into general outpatient departments or non-communicable disease (NCD) clinics [40], community-based ART distribution strategies [41–43], and extended scripting of ART [44–46]. Further research is needed on feasible and scalable confidential and/or community-based strategies for ART initiation.

Health care workers acknowledge that ART initiation and retention among healthy clients disrupts routine, daily responsibilities essential for clients to maintain normalcy. Clients are expected to visit a health facility every month for the first 6 months after initiation [21]. Frequent visits are compounded with long wait-times [47,48]. This creates competing demands for healthy clients who are likely not exempt from standard social norms and obligations that also demand extensive time and energy. Improving facility efficiencies, including reduced waiting times and early morning or after-hours options could reduce barriers for healthy clients. Differentiated models of care for initiation and retention among clinically stable, healthy clients may be warranted to address conflicting demands between clients’ clinical and social obligations.

HCWs believe healthy clients are often not ready to start ART because of limited knowledge about the benefits of early ART initiation. Similar findings have been reported in other sub-Saharan countries [32,33]. Community sensitization regarding the benefits of early ART may help transform the current dogma of HIV treatment. Treatment as prevention messaging may be particularly motivating to healthy clients since it provides immediate benefits for early initiation as opposed to future benefits, which may be abstract and more difficult to comprehend [49].

HCWs believe healthy clients need more time to accept their new HIV status. While HIV is now a manageable, chronic illness [50,51], a positive diagnosis is still seen as disrupting social order and norms [32]. Like other life-altering illnesses, clients may need time to grieve before they can accept treatment, especially clients who are still healthy and therefore are not motivated for treatment by a desire to return to health [52–54]. To address these concerns, a chronic care model [55] could be adopted that promotes shared decision-making between provider and patient, provides resources for clients to come to terms with their diagnosis, and links clients to community-based resources that help clients regain normalcy [55–58].

Finally, side effects remain a concern. Side effects are often present early after initiation of efavirenz-containing regimens, which are still first-line in many resource-limited settings, including Malawi [21]. While many side effects are short-lived and mild, some can persist and be debilitating, particularly mood changes and sleep disturbance [59]. These side effects may be extremely difficult for healthy clients, while ill clients may be more likely to endure side effects in order to achieve improved health following AIDS-related illnesses [60]. Concerns around side effects may be reduced by the planned rollout of dolutegravir as first-line ART in Malawi, as this antiretroviral has an improved tolerability profile compared to efavirenz [61].

## Limitations

This study has several limitations. First, we rely on a sample of HCWs from one region of Malawi, limiting the potential generalizability of findings. Second, we rely on perceptions from HCWs, and it is unclear the extent to which clients agree with study findings. Finally, based on the qualitative nature of the study, we are unable to test the association between described barriers and actual uptake of ART.

## Conclusion

The potential benefits of universal Test and Treat cannot be reached unless healthy populations are motivated to initiate ART and remain in care. Feeling health was seen by HCWs to exacerbate barriers to ART initiation, and resulted in limited motivation among healthy clients. In order to achieve high levels of ART uptake and retention, client perceptions of treatment benefits must outweigh perceived risk within both social and physical domains [14]. Innovative strategies within the heatlh system are needed to address potential heightened barriers experienced by healthy clients. In particular, innovative service delivery models are needed to ensure privacy and time efficiency within health facilities, as well as extending HIV services outside facility settings.

## Paper context

Universal Treatment for Antiretroviral Therapy (ART) is rapidly spreading throughout sub Saharan Africa. Despite its potential benefits, very little social science research has examined how the policy will be interpreted on the ground particularly among healthy HIV-positive clients. Before Test and Treat, healthier clients were less likely to initiate ART. This may continue unless we examine the unique barriers to care experienced by this population. We address an important question: ‘What factors influence ART initiation among healthy HIV-positive clients?’
